# Integrative Review of the Recent Literature on Human Resilience: From Concepts, Theories, and Discussions Towards a Complex Understanding

**DOI:** 10.5964/ejop.2251

**Published:** 2022-02-25

**Authors:** Clément Métais, Nicolas Burel, Jane E. Gillham, Cyril Tarquinio, Charles Martin-Krumm

**Affiliations:** 1 EA 4360, APEMAC - UDL, Metz, France; 2 F3S, University of Strasbourg, Strasbourg, France; 3 Teaching and Research Unit in Physical Education and Sport (UER-EPS), University of Teacher Education, Lausanne, Switzerland; 4 SENS-EA.3742, Université Grenoble Alpes, Grenoble, France; 5 Psychology Department, Swarthmore College, Swarthmore, PA, USA; 6 Laboratoire de Psychologie de l'Ecole de Psychologues Praticiens, Paris, France; 7 IRBA, Brétigny, France; Connection Lab, San Francisco, CA, USA

**Keywords:** resilience, ecological approach, transactional approach, integrative methods

## Abstract

Resilience may be viewed as the capacity of an individual, or perhaps of a dynamic system, to adjust and adapt positively to adversities and disruptions that impact one’s functioning and development. Yet a common statement in the literature is that there are still today numerous ways of defining and conceiving resilience. This multiplicity of approaches calls for clarification and generates a need of common theoretical ground. Therefore, this review aims to examine, to clarify and to synthesize how “human” resilience is conceptualized within the recent human sciences literature to help answer the question: ‘What are the key approaches, concepts, and definitions of resilience?”. Following Whittemore and Knafl (2005, https://doi.org/10.1111/j.1365-2648.2005.03621.x) methods, an integrative review of the recent resilience literature (2013–2019) was undertaken. Four databases were used for the search: PsycINFO, PubMed, ERIC, Google Scholar. A reference and citation tracking was then performed on the papers identified. Sixty-nine papers passed all the stages (identification, screening, eligibility, inclusion) and formed the sample. Results show that resilience definitions are nowadays either about “adapting and bouncing back to previous levels of health” or about “thriving and rising above the adversity towards increased levels of health.” Results also show that resilience features—antecedents, mechanisms, consequences—are mainly conceptualized in a vertical sequence where an antecedent influences another or influences a mechanism leading to consequences. This paper concludes that modern conceptions can fit within a transactional and constructivist approach that goes beyond the former approaches by providing a more nuanced and realistic picture of the resilience process and by viewing it as a dynamic and person-situation-defined process.

## Resilience Throughout the Years

Owing its inception to material sciences, the term resilience has been broadly used in many disciplines to forward change, disruption, adaptation, or to illustrate flexibility and sustainability (Scoloveno, 2016; Sivilli & Pace, 2014; Xu & Kajikawa, 2017). When studying such a construct it should always be remembered that resilience is much about the consideration of *adversity* and *positive adaptation*, these two dimensions being inherent to the study of resilience (Fletcher & Sarkar, 2013; Luthar, Cicchetti, & Becker, 2000; Luthar, Crossman, & Small, 2015; Masten, 2015; Wright, Masten, & Narayan, 2013). In psychology, although resilience is a broadly studied topic, the large amount of published work about this construct still generates a lack of consensus (Ayed, Toner, & Priebe, 2019; Christmas & Khanlou, 2019; Panter-Brick, 2014; Park & Kim, 2014; Ungar, 2008; Vella & Pai, 2019). The multiplicity of definitions, approaches, and theoretical orientations beclouds all the knowledge gained (Masten, 2018) and calls for clarification (Christmas & Khanlou, 2019; Fletcher & Sarkar, 2013; Fogarty & Perera, 2016; Leys et al., 2018; Luthar et al., 2000; Rasmussen et al., 2019). As such clarification is essential in shaping a substantial and coherent basis for practical applications and health policies (Ayed et al., 2019; Christmas & Khanlou, 2019; Leys et al., 2018), the major goal of this paper is to examine and synthesize the more recent approaches to resilience. Its novelty is first to provide such a clarification by performing an integrative review (Whittemore & Knafl, 2005) of human resilience within the recent (2013–2019) human sciences literature, and second to extend its overall discourse by discussing about an innovative transactional and constructivist approach subjective to situations so that it accounts for contextual, inter-individual, and developmental differences.

Although progression might not have been streamlined as hereafter, four great eras can yet be distinguished within the five or so decades that saw the rise and evolution of resilience research (Luthar et al., 2000; Masten, 2007, 2014, 2018; Wright et al., 2013).

It started in the ‘50s and ‘60s with the field of mental health—quite dominated by disease-oriented models—being the playground for studies focusing on mental illness problems and on significant threats to development (Luthar et al., 2000; Masten, 2007; Wright et al., 2013). It is at that time the term resilience was brought to replace the term “invulnerability” used to describe children who were thought to be impervious to adversity (Luthar et al., 2000; Wright et al., 2013). During this first era, the purpose was to define and to understand resilience. Observations led to noticing differences between individuals’ adaptation (positive or negative outcomes); these were ascribed to internal psychological resources such as personal abilities and strengths (Garcia-Dia, DiNapoli, Garcia-Ona, Jakubowski, & O’Flaherty, 2013; Luthar et al., 2000; Masten, 2007; Wright et al., 2013). The second era, since the late ‘70s and ‘80s, brought clarification and induced a more ecological understanding of the construct, arguing that external factors were also extremely important so that resilience, rather than a trait, should now come to be understood as a process implying internal and external influences. This ecological broadening progressively saw a shift from the “‘*what*’ questions of description to the ‘*how*’ questions of underlying processes that influence adaptation” (Masten & Best, 1990, p. 439) and led the way towards the third era (‘90s, 2000s) of the field. Progress on the theoretical understandings of the resilience functioning allowed researchers from this wave to develop and experiment with interventions to foster well-being such as preventive resilience-based interventions (Masten, 2007). Finally, the modern and fourth era (2000s through the present) of resilience is about integrating understandings of internal and external resources with knowledge from a variety of disciplines (e.g., genetics, neurobehavioral development, or mathematics with the use of statistical analysis) to fully grasp the complexity of this construct and to bring our comprehension a step further (Liu, Zhang, Ji, & Yang, 2018; Shi, Sun, Wei, & Qiu, 2019; Shrivastava, De Sousa, & Lodha, 2019; Van der Werff, van den Berg, Pannekoek, Elzinga, & van der Wee, 2013). This modern era is said to be integrative and holistic (Christmas & Khanlou, 2019; Vella & Pai, 2019), so that it combines all the previous findings since the birth of resilience research, with technical progress enabling better physiological understandings and more realistic outcomes assessment, and with the growing influence of multidisciplinary knowledge (Masten, 2007).

Throughout the evolution of the resilience construct, three central questions seemed to raise debates, and to feed the critics between divergent theoretical approaches.

## Is Resilience Based on Internal or External Factors?

The traditional approach and early conceptions were viewing resilience as a binary or dichotomous inner property as the individual was considered having the internal psychological dispositions to face adversity, or simply not having them (Anthony, 1974). An individual was then deemed resilient or not, only by assessing an absence or presence of psychopathology and disorder within him or her upon experiencing adversity. Although such former acceptations viewing resilience as the absence of disorder amount to saying health could solely refer to the absence of disease (Almedom & Glandon, 2007), an *advantage* of the traditional approach is the recognition that people can have internal cognitive resources and coping skills that are helpful in overcoming adversity. These findings greatly helped increase the understanding of the profile and of the internal characteristics of those being resilient, those that were able through resilience to meet homeostasis needs (i.e., getting back to an equilibrium, to normal functioning). Prince-Embury (2014) summarizes the main personal qualities (internal factors) that were believed to be the only ones leading to resilient outcomes; those were intellectual ability, easy temperament, autonomy, self-reliance, communication skills, and effective coping strategies.

On the other hand, the lack of consideration about the fact human beings are evolving in an environment with external influences, can be considered as a *disadvantage* as the traditional conception of resilience only draws a partial explanation of what truly happens when someone is confronted to adversity.

Though this approach has given place today to a more ecological and dynamic understanding of resilience encompassing the influence of external factors (Fritz, de Graaff, Caisley, Van Harmelen, & Wilkinson, 2018; Luthar et al., 2000; Masten, 2007; Wright et al., 2013). Ungar and Liebenberg (2013) advanced that the emphasis on personal assets might be beneficial, but that on an ecological point of view “the environment accounts for far more of the developmental gains of a population at risk than individual factors” (Ungar & Liebenberg, 2013, p. 246). The definition of resilience within such an ecological approach would hence be about “the capacity of a dynamic system [the individual] to adapt successfully to disturbances that threaten system function, viability, or development” (Masten, 2014, p. 6) or about processes that predict developmentally appropriate outcomes in the face of threats to development (Kaplan, 1999; Masten, 2001). Anyhow, in a situation where an individual is experiencing adversity (chronic or acute) and then engages in an adaptative process, we can concretely distinguish both deleterious factors and factors that foster positive outcomes. Since distance has been taken from the primary focus on the inner qualities of the resilient child, ecological understandings led several researchers to sort out those factors according to three main categories encompassing both internal and external features: 1) children’s (or any individual) personal attributes, 2) aspects and attributes of their families such as secure and caring relationships (ideally), and 3) broader external influences from their social environment such as social relations with peers, school, or the wider community (Anaut, 2015; Luthar et al., 2000; Masten, 2014; Masten & Garmezy, 1985; Werner & Smith, 1992).

## Is Resilience a Static or Dynamic Construct?

Quite analogous and complementary to the question of resilience being related to internal or external factors, another central question is whether to view this construct as an internal trait or as a process (Southwick, Bonanno, Masten, Panter-Brick, & Yehuda, 2014). These concerns in the end, may both lead towards asking more generally whether to consider resilience in a static or in a more dynamic fashion. Although quite authentic, this interrogation seems yet unanswered and is still often debated in the current literature (Leys et al., 2018; Liu et al., 2018; Rolin, Fossion, Kotsou, & Leys, 2018; Shrivastava et al., 2019; Sisto et al., 2019).

Solely relying on internal factors and psychological traits accounts for saying that resilience is a personality characteristic stable over time (Block, 1995). This was common sense in the early eras of resilience, where such a construct—termed *ego-resiliency*—would then be defined as someone’s ability to adapt its emotion control up or down depending on the situation (Block & Block, 1980). Although nowadays a few researchers still relate to resilience as a trait (e.g., Kalathil et al., 2011; Taormina, 2015), much of these traditional understandings were rapidly declared partial and research moved towards the perspective of dynamic processes, so that today there is a growing consensus against the use of resilience as a fixed and stable trait (Craciun, 2013; Infurna & Luthar, 2018; Liu et al., 2018; Luthar et al., 2000, 2015; Malhi, Das, Bell, Mattingly, & Mannie, 2019; Masten, 2014, 2015; Masten & Barnes, 2018). In this perspective, the ecological approach is a first reaction to the limits of the traditional approach, so that resilience is seen as a “developmental process or a dynamic capacity rather than as a static outcome or trait” (Yates, Tyrell, & Masten, 2015, p. 773).

Researchers have also highlighted two types of underlying processes, that is, compensatory processes and moderating processes. On the one hand, *compensatory processes* are associated with factors that have neutralizing or counteracting effects regardless of the adversity level. In this first category, *resources*—also known as promotive factors (Sameroff, 2000), or assets—compensate or neutralize the effects of adversity (e.g., intellectual ability, extraversion), whereas *risk factors* act against positive adjustment or might precipitate the occurrence of adversity itself (e.g., childhood maltreatment, neglect). On the other hand, *moderating processes* are associated with factors that have effects varying in regard to the level of adversity. In this second category, *vulnerability factors* exacerbate the already ongoing negative effects of adversity (e.g., absence of health-care increasing the negative effects of one being ill), whereas *protective factors* are moderators that temper negative effects of adversity, or show stronger salience in high levels of adversity (e.g., supportive teacher-pupil relation being particularly effective when the pupil encounters problems) (Luthar et al., 2015; Masten, 2014; Prince-Embury, 2014; Rutter, 2013; Wang, Zhang, & Zimmerman, 2015; Wright et al., 2013; Yates et al., 2015).

## Does Resilience Consist of Bouncing Back or Bouncing Forward?

As mentioned earlier, it is important to consider resilience both upon adversity and positive adaptation such as clearly displayed in the definition proposed by Yates et al. (2015) where resilience pertains to “the processes by which individuals achieve positive developmental outcomes despite exposure to known threats to adaptation” (Yates et al., 2015, p. 773). Yet does this positive adaptation mean bouncing back to previous homeostasis and well-being levels (i.e., pre-adversity healthy functioning levels), or does it rather relates to an individual thriving towards increased levels of health (i.e., bouncing forward)—which would amount to using adversity as an opportunity for growth (e.g., post traumatic growth; see Tedeschi et al., 2018)?

To this question, the encounter of resilience research—mainly influenced in its evolution by developmental psychology—with clinical psychology brought a fresh momentum and offered a distinct and more nuanced perspective on the matter. In fact, developmental research on children’s resilience and clinical psychology studying adults, have grown in two separate ways (Infurna & Luthar, 2018), whereas they could have gained insights from one another. Nowadays, as opposed to the field of developmental psychology which focused largely on the “gradual emergence of signs of favorable adjustment” to *chronic* (i.e., ongoing, long-lasting) *adversity* (Bonanno & Diminich, 2013, p. 380), several trauma researchers (e.g., Bonanno, 2004; Bonanno & Diminich, 2013; Galatzer-Levy, Huang, & Bonanno, 2018) focus now on adults’ positive adaptation after experiencing a potentially traumatic event (PTE)—that is, *acute adversity* or a specific stressor occurring at a given time (Bonanno, 2004). Other research confirms that adversities can either be chronic or acute (Masten, 2014; Masten & Narayan, 2012; Noltemeyer & Bush, 2013; Yates et al., 2015). Additionally, advances in statistical methods such as growth curve modeling enabled Bonanno and Diminich (2013) to highlight several outcome trajectories following this acute kind of adversity: *Chronic dysfunction*, *delayed dysfunction*, *recovery* (i.e., gradual emergence of positive adaptation), and *minimal-impact resilience* (i.e., rapid emergence of positive adaptation). Conducive to the full consideration of individual and contextual differences, these trajectories have somehow grown to a consensus so that a trajectory approach became quite accepted (Galatzer-Levy et al., 2018) and has even spread to the developmental field (Masten, 2018).

Moreover, although in the last decade resilience research moved forward thanks to new subfields of psychology—such as research on resilience in the workplace (e.g., Vanhove, Herian, Perez, Harms, & Lester, 2016)—its encounter with positive psychology came to be quite significant as well. In the late ‘90s and early 2000s, Seligman and Csikszentmihalyi argued on the urgent need for rethinking psychology and creating a field of positive psychology that aims at enhancing the positive side of psychology rather than the traditional focus on pathology (Seligman, 2000; Seligman & Csikszentmihalyi, 2000). Akin to the clinical field, positive psychology offered interesting new perspectives such as the promotion of well-being in treatment therapies, in school-based programs (positive education, resilience programs), or when facing lighter adversities of the every-day life. Although such an encounter came to be relevant first in terms of field applications and interventions, positive psychology—by its emphasis on well-being promotion—also gave more weight to a “thriving” and “bouncing forward” orientation of the resilience process (Schwarz, 2018).

Yet even today questions about the orientation to be given to the resilience process and outcome have not been properly resolved. Many researchers in their studies argue that bouncing back is a key component of resilience and several authors support the idea that resilience is about recovering from the negative effects of adversity and returning to the standard base level of health and wellbeing (Aburn, Gott, & Hoare, 2016; Dowrick, Kokanovic, Hegarty, Griffiths, & Gunn, 2008; Edward, Welch, & Chater, 2009; Felten & Hall, 2001; Garmezy, 1991). Others suppose that “perhaps it is better to conceptualize resilience as a process of moving forward and not returning back” (Southwick et al., 2014, p. 3). This makes sense if we consider existence as a developmental continuum, where every experience brings the individual a step forward on his or her developmental path. This design is also shared by Cyrulnik and Duval (2006) via the idea of a “neo-development” as the outcome of the resilience process (Anaut, 2015, p. 36).

## Method

### Research Question

As per the aims exposed earlier, the primary research question was: *What are the key approaches, concepts, and definitions of resilience within the recent literature?*

### Protocol

Integrative methods described by Whittemore and Knafl (2005) were used in order to capture a clear picture of our topic and to draw conclusions. These methods enabled the inclusion of theoretical as well as methodological papers. The process of this review followed five stages: Problem identification, literature search, data evaluation, data analysis, and presentation—which is a display of the results (discussions, tables, and figures of this paper).

### Information Sources

The following databases: Google Scholar, PsycINFO, PubMed, and ERIC, were used for the search focusing on the recent literature about resilience (i.e., search covering a 7-year period: 2013–2019). All the records went through the PRISMA stages, that is, identification, screening, eligibility, and inclusion (Moher, Liberati, Tetzlaff, Altman, & Group, 2009).

### Search Strategy

The following key words were searched: *(English terms)* resilience review, resilience concept, resilience concepts, resilience construct, resilience theory, resilience adversity, resilience science, resilience *and* [reply *or* debate *or* discussion], resilience perspectives; *(French terms)* résilience concept, résilience concepts, résilience revue, résilience principe, résilience définition, résilience théorie. These key words were searched in the title of the papers. The main reviewer being French, the French equivalents of the English terms were searched to provide extra valuable references to be potentially added to the review sample. No other languages were targeted as the authors do not speak more than French and English.

### Study Records and Criteria

A total of 2,776 records were found after the initial search. These records were *screened* by title and abstract, and out of this stage 1,079 papers were excluded because relating to irrelevant sorts of resilience (e.g., ecology, finance, physics, policies and organizational studies). The remaining 1,697 papers relating to “human” resilience were kept and transferred into a reference management software able to manage bibliographic data. As well data of the “search history” was registered by the means of a spreadsheet software.

At the beginning of the *eligibility* stage papers were included if they focused on human psychological resilience (i.e., general approaches, theories, concepts), or if they contained section(s) on general approaches, theories, and concepts of resilience. At this point, 1,490 papers relating to specific domains, specific topics, specific contexts, specific subjects involved—for example diabetes patients, cyberbullying, LGBTQIA2+ populations (i.e., lesbian, gay, bisexual, transgender, transexual, queer, questioning, intersex, asexual, allies, pansexual, others), geriatrics, ethnic systems, racism, family violence, cancer survivors, etc.—were excluded of the review, and 207 papers were retained because of being either general papers on human psychological resilience (i.e., general approaches, theories, concepts), or other papers containing section(s) on general approaches, theories, and concepts of resilience. Then in order to complete this stage, 98 duplicates were finally removed from the retained papers.

However, the inclusion criteria exposed here above should not be connoted to a lack of awareness. Specific or targeted papers contribute to the knowledge of resilience, and for sure every psychological, emotional, or social constructs/processes occur in a specific context. We did not intend to strip resilience of any meaning; our focus here was rather about grasping general theoretical patterns of resilience. We looked for papers displaying an overview of the field, general understandings and patterns of resilience applicable to several contexts, current debates and discussions, as well as future perspectives of the field.

Ultimately, out of the 109 eligible papers 27 were excluded for being still not general enough (i.e., those ones having passed *eligibility* because containing sections on general approaches and theories of resilience but then eventually considered too specific in this more selective ultimate stage; for example, contexts and topics on firefighters, sport athletes, medical employees, post TBI therapy, homeless people), and 13 full-text PDFs could not be gathered (i.e., not found on the internet, then not accessible via the reviewers’ French university web-library access, then no positive answer to the requests sent to authors). A total of 69 papers were included in the final selection for the integrative review. The review process was performed by the main author; yet, inclusion processes and final selection were supervised and assessed by two other reviewers (second and last authors). A great majority of the papers included in the review stem from psychology literature, but a few pertain to the fields of medicine, nursing, sociology, neuroscience, and sustainability science (*cross-field domain*). The sample includes a few book chapters (9), book review (1), plenary panel and conference reports (2), published thesis work (2), scholarly report paper (1), but most of the papers are journal articles (54). These journal articles are mainly theoretical articles; only a few are literature reviews, concept analysis or systematic reviews. Empirical articles have not been avoided on purpose, the ones found in the search were very context-subject-situation-specific and did not fit our criteria.

**Figure 1:**
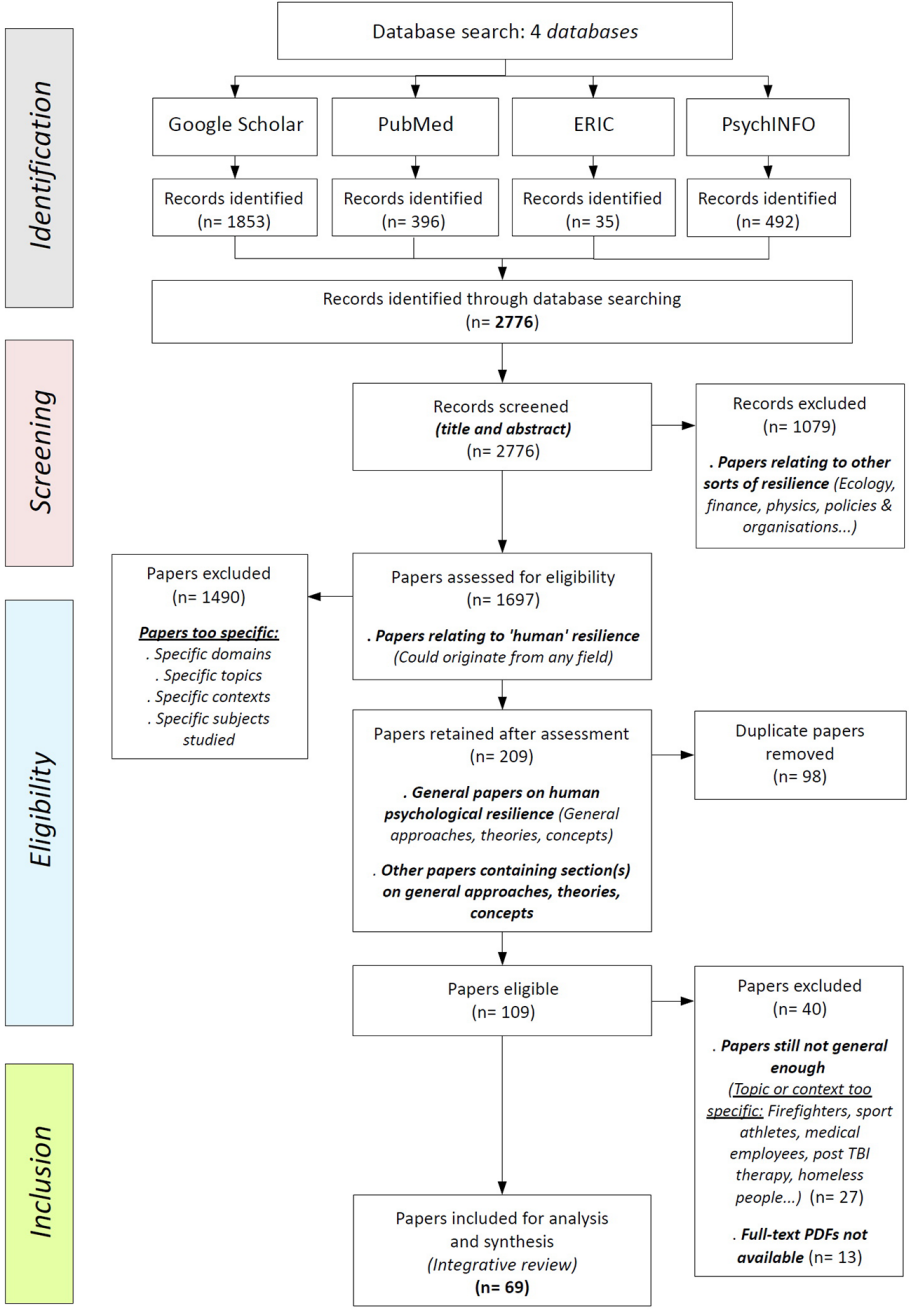
Study Flow Chart

Inclusion criteria, search words, and a list of the papers are available in the APPENDIX (see Tables A1–A3), part of the Supplementary Materials of this paper. Flow chart (see Figure 1) is presented here below.

### Extra Theoretical Ground—Landmark Papers

Throughout our readings (i.e., final literature sample of the present review and cross-reference readings) two papers (Luthar et al., 2000; Masten, 2007) were regularly cited and stood out before undertaking any process of coding and data extraction.

Having in mind that our primary focus is the recent literature about resilience, it should be noted that—although not included in our sample—both of these papers from the previous decade really helped inspire and organize our reflection and the structure of this paper. They confirmed our ideas about some shift in the evolution of resilience research from quite traditional paradigms to more ecological paradigms. Thereby, we came into coding already cognizant of traditional versus more ecological and complex approaches to resilience. Attention was paid to this distinction through the coding and data extraction processes.

### Data Selection Process

First, definitions of resilience were extracted from each paper and registered as well via a spreadsheet software. Although it seemed appropriate to give credit to the whole selection no matter the weight/strength of the paper it remained important to extract and specify the source of the definitions (author’s own/from other author). Of the 69 papers included in the review, nine (13%) did not present a clear definition of resilience, but 60 (87%) provided one. Note that out of those 60, 36 (60%) displayed the author(s)’ own definitions, whereas 24 (40%) presented definitions from other authors.

In a second step, features of resilience were extracted from each paper and registered via a spreadsheet software. Thereby every term referring to factors influencing resilience, to some attributes, or consequences were included and extracted. Of the 69 papers included in the review, 12 (17%) did not provide any data regarding resilience features, but 57 (83%) provided some pertaining to features of the ecological conception of resilience.

### Data Items

The definitions of resilience retrieved from the papers were sorted into the following subgroups that the authors came up with as they thought such groups would illustrate well the definitions’ theme and orientation: Adapting and maintaining health and functioning levels; Adapting and bouncing back; Adapting (no specificity added); Adapting towards positive development (rising above, thriving, growth); Mix of previous categories.

On the other hand, features of resilience could have been clustered in accordance to the Walker and Avant process of concept analysis highlighting antecedents, attributes, and consequences of a construct (Walker & Avant, 2011). Yet, following a discussion between the first two authors and the last author, it was decided to adopt a slightly refitted sequence—that is, antecedents, *mechanisms*, and consequences. Contrastingly to Hartmann, Weiss, Newman, and Hoegl (2019) highlighting—in their paper on resilience in the workplace—the importance of studying resilience mechanisms, the recent literature does not significantly highlight the features relative to what truly happens during resilience. That is, regularly, when mentioning resilience attributes—that is, core functioning features of resilience—authors tend to refer to features influencing resilience, rather than real mechanisms or processes occurring in the context of this successful adaptation. How does resilience really work? Step by step, what occurs? What are the mechanisms triggered/activated? These concerns should be paid true attention as the ‘*how*’ question about resilience is crucial in terms of field applications and health strategies (Christmas & Khanlou, 2019; Mc Gee, Höltge, Maercker, & Thoma, 2018).

Therefore, antecedents, *mechanisms*, and consequences were preferred to the Walker and Avant categories (Walker & Avant, 2011). All of the terms or resilience features referring to mechanisms or processes illustrating how resilience works and to what truly occurs were then clustered into the ‘*mechanisms*’ array, whereas some features such as self-esteem (Scoloveno, 2016) or self-efficacy (Garcia-Dia et al., 2013)—often categorized as attributes in the recent literature—were re-categorized as *antecedents*. These examples—self-esteem, self-efficacy, and the like—are not mechanisms or processes, they are rather pieces defining the individual, something that she or he “*has in* store,” standing as a trait or as a skill.

### Data Synthesis

As mentioned before, the definitions were organized by theme categories. Yet the authors found that such categories could be more broadly illustrated by two ideas reflecting the two orientations taken by resilience definitions in the recent literature: *Adapting and bouncing back to previous levels of health and development*, versus *thriving and rising above the adversity towards increased levels of health*.

Similarly, features of resilience were more accurately repacked: Antecedents were divided into “internal supportive features” (*physical states, character traits, and cognitive, social, and emotional states*) and “environmental (external) supportive features” *(family, social cluster, broader external systems/clusters, inanimate features, others*), whereas mechanisms were divided into “coping, mentalization-coherence, empowerment” sub-groups, and consequences into “homeostasis purpose” or “growth purpose”.

Regarding this categorization, the chosen groups and categories were not set prior to the coding process. The authors did not start coding with these particular traits, states, mechanisms’ sub-groups, and consequences in mind; it is rather through the process of coding that these appeared to categorize well the data extracted. More precisely, “*character traits*” were additionally divided according to the “*big five personality traits*” theory (Costa & McCrae, 1992)—*openness, conscientiousness, extraversion, agreeableness, neuroticism*. Note that the use of the “*big five personality traits”* here does not mean only the data pertaining to those traits were extracted. Data extraction did not take credit of this classification; it is only afterwards that character traits were repacked according to this well-established model. Furthermore, most of the features clustered in the “*states*” categories might appear rather like skills. Though, the use of the label “states” is based on the fact these features are resources and skills that might be temporary, and that can be improved or lessened across lifespan. Distinction between *traits* versus *states* seemed interesting to us. Regarding the mechanisms of resilience, proper definitions of the *coping*, *mentalization-coherence*, and *empowerment* concepts will be presented further in this paper.

## Results

### Characteristics of the Ecological Approach

#### Divergent Orientations in the Acceptation of Resilience

As an illustration to the unresolved questions about the orientation to be given to the resilience process and outcome, a great variability within the resilience definitions appears to stem from the recent literature on modern and ecological approaches to resilience.

**Figure 2:**
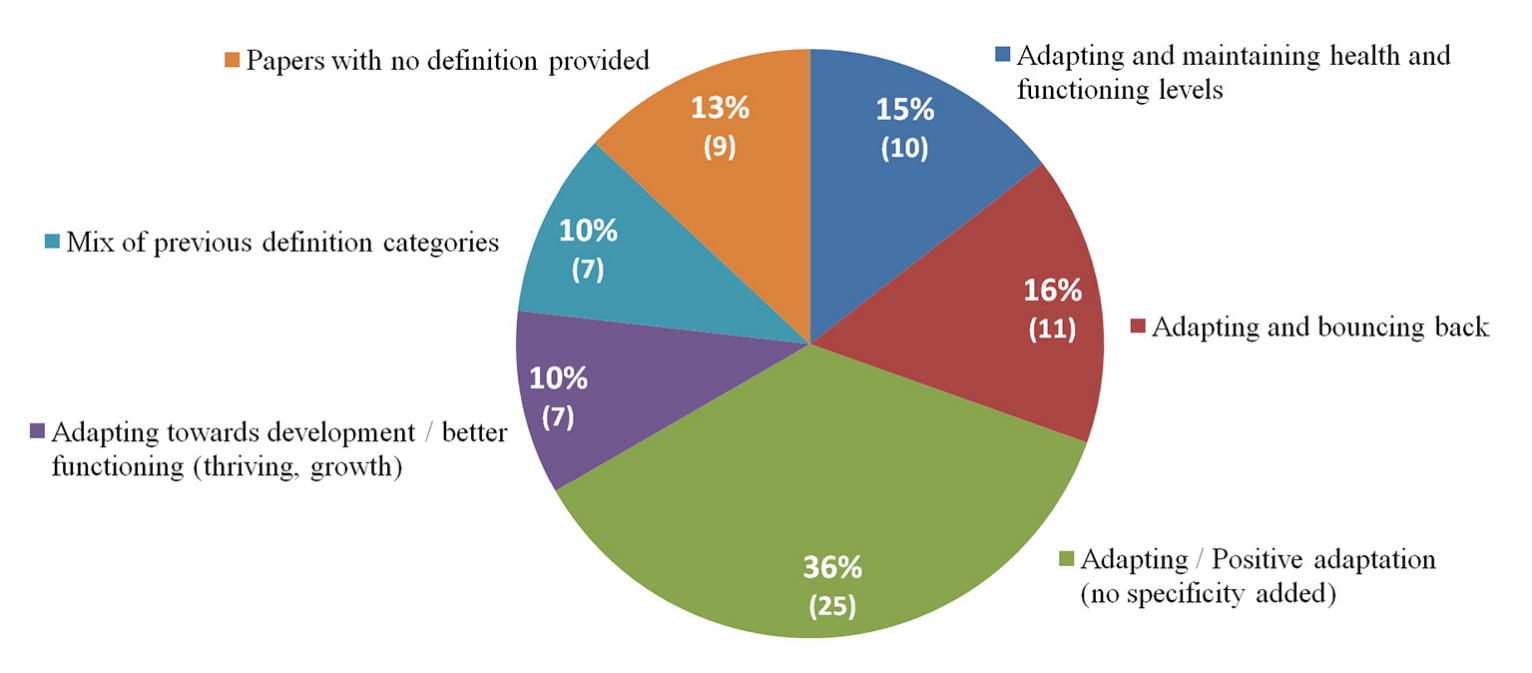
Theme Categories of Resilience Definitions

Illustrated below (see Figure 2) are the definitions of resilience gathered in the review sample. As mentioned earlier these were sorted into five categories: Adapting and maintaining health levels; Adapting and bouncing back; Adapting (no specificity added); Adapting towards positive development (rising above/thriving); Mix of previous categories. Somehow, these categories reflect two broader directions that resilience could take: *Adapting and bouncing back to previous levels of health and development* versus *thriving and rising above the adversity towards increased levels of health*. These two broader directions induced by the categories of the definitions confirm that no consensus has yet been found upon whether resilience is about bouncing back or bouncing forward. Raw extracted data on resilience definitions is available upon request from the first author.

#### Features of the Ecological Approach—Antecedents, Mechanisms, Consequences

**Figure 3:**
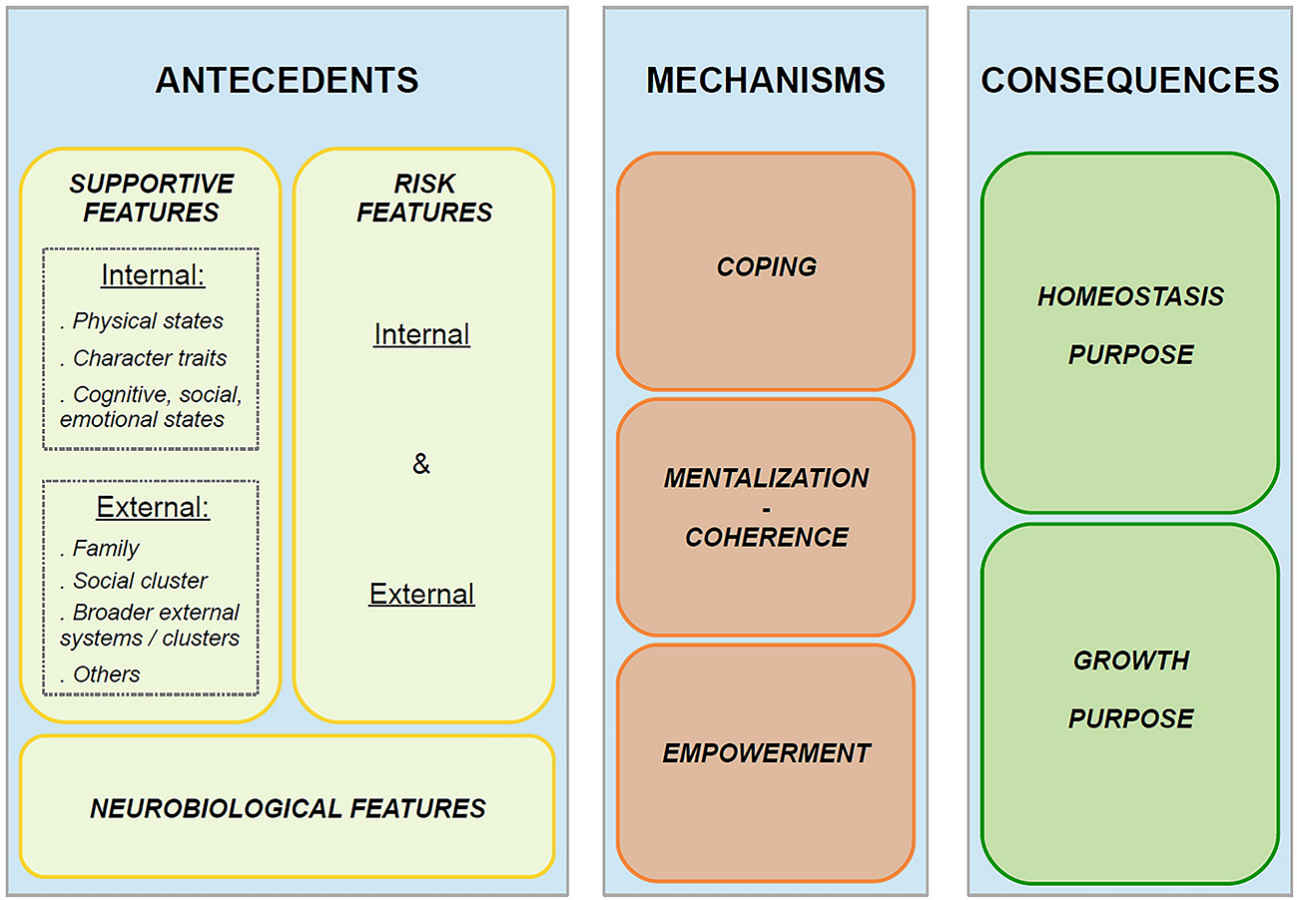
Ecological Approach—Overall Display of the Resilience Features

Here below (see Figure 3) is a condensed and simplified illustration of the different categories and great features that revolve around the construct of resilience. For an exhaustive and detailed view of the features, please refer to Tables 2a and 2b available as Supplementary Material and in which these features extracted from the review sample are all listed and clustered. Raw extracted data about resilience features is available upon request (from the first author).

As detailed previously in the methods section, the “antecedents, *mechanisms*, and consequences” features classification was preferred to the Walker and Avant classification (Walker & Avant, 2011). More precisely, antecedents were divided into “internal supportive features” (*physical states, character traits, cognitive and socio-emotional states*) and “environmental (external) supportive features” *(family, social cluster, broader external systems/clusters, inanimate features, others*). Consequences were divided into “homeostasis purpose” and “growth purpose”. Similarly, as discussed between the authors and according to the concepts exposed in the paragraph below, “coping, mentalization-coherence, empowerment” were thought to illustrate well sub-groups into which the extracted features pertaining to mechanisms of resilience could be clustered.

The term “*coping*” comes from Lazarus and Folkman (1984) with their cognitive appraisal theory. It corresponds to the “constantly changing cognitive and behavioral efforts to manage specific external and internal demands that are appraised as taxing or exceeding the resources of the person” (Lazarus & Folkman, 1984, p. 141). Through different appraisals (i.e., *first appraisal* concomitant with the detrimental experience and *second appraisal* occurring later) the individual becomes aware of those taxing demands begotten by the adversity encountered. Therefore coping usually occurs during the adversity or in the aftermath of this adverse experience so that the individual may deal with and reduce the resulting stress. It is a process that also enables the development of one’s first narrative of the experience lived. Yet let us note that although appraisal theories have been largely supported, other theories—such as the Conservation of Resources (COR) theory (Hobfoll, 1989, 2001) upon which the stress produced by adversity is rather due to the objective loss of resources than due to the subjective appraisals of the adversity (Hobfoll et al., 2007; Johnson et al., 2007)—have started to show interest and bring nuance in conceptualizations of stress processes.

The term “*coherence*” (*sense of*) was formulated by Antonovsky in 1979. It relates to the anticipation of events—by the means of believing in the predictability of the environment—and to the analysis and synthesis of particular situations in order to avoid detrimental outcomes (Antonovsky, 1979). More simply, it could be related to a process of meaning-making, or *mentalization*, (i.e., standing back to re-interpret and reflect on a past or an on-going adverse situation, in order to build up alternative narratives). The mentalization/coherence process in the case of acute adversity could occur some while after the experience of adversity as the individual needs time to assimilate the adverse experience in order to reflect on it and to start building up intentions to set goals of “*doing better*.” In the context of a more chronic adversity, this process could occur also during the adverse experience itself (e.g., dealing with ongoing stress at work, or dealing with a loved one’s serious illness).

Finally, the term “*empowerment*” was introduced by Rappaport (1987). It pertains to a process by which the individual—through concrete actions, with the help of relational support, or by the means of his agency on the environment—socially and psychologically adjusts and adapts (Le Bossé & Lavallée, 1993; Rappaport, 1987). More recently, empowerment was defined as “a meaningful shift in the experience of power attained through interaction in the social world” (Cattaneo & Goodman, 2015, p. 84), where power is “one’s influence in social relations at any level of human interaction, from dyadic interactions to the interaction between a person and a system” (Cattaneo & Chapman, 2010, p. 647). In fact, sometimes meaning-making through coherence/mentalization mechanisms is not enough. Turning this around towards action-growth—*doing what is meaningful*—should be an additional goal in order to adapt positively (Hobfoll et al., 2007). Such a perspective is particularly relevant when considering post-traumatic growth (PTG). That is, when not associated with proper action (which can as well be in the mind, i.e., facing and challenging our thoughts and emotions) from the individual then PTG is not necessarily corollary of well-being and can rather be related to more distress (Hobfoll et al., 2007; Johnson et al., 2007). Therefore in the present review, empowerment was used to categorize the mechanism by which an individual has some kind of extroversion intention and fulfils it with proper actions, such as seeking social help, sharing narratives of the adverse experience to better bounce back from it, or even one taking control of his or her agency/power on the surrounding system (e.g., setting new resolutions, putting on some fresh and constructive mindset to work better). Empowerment processes could occur a little while after mentalization/coherence when the individual is ready for extroversion, or it could occur soon after the experience of adversity to foster coping processes, yet it may also take place later and throughout time to enhance mentalization. More generally, it should be noted these three mechanisms (coping, mentalization/coherence, empowerment) are iterative processes; they can be concomitant and enhance or even trigger each other.

### Limits of the Ecological Approach

#### Limits Arising From the “Cripping” Critics

Although resilience research has since a while moved towards the inclusion of external factors, the current ecological conceptions still have their missteps and still generate a lot of pressure and expectations on the inner capacities of the individual. In fact, modern conceptions of resilience are quite immersed in a western normative point of view—that is, standardized western conceptions of resources, strengths, and adaptation to adversity, where an individual is expected to show competence (Fletcher & Sarkar, 2013; Hutcheon & Lashewicz, 2014; Hutcheon & Wolbring, 2013; Schwarz, 2018; Ungar, 2008; Ungar & Liebenberg, 2011; Wright et al., 2013). This could even lead to the term “*cripping*” resilience, as today’s definitions “are plagued by hegemony in that they align with western, middle-class, ableist norms of healthy, normal, or valued functioning” (Hutcheon & Lashewicz, 2014, p. 1387). This represents a real discriminating issue when such standardization might legitimate as “aberrant behaviour” the responses of disabled people, of people categorized as impaired and who do not fit in these norms, or simply of those showing particular functioning (Schwarz, 2018, p. 530). Added to that, the *commodification* processes—that is, goods, ideas, and skills established as tradable and as having commercial value (Timimi, 2017)—and the division between sick and healthy, or vulnerable and resilient, altogether reinforces the reproduction of social imbalances in a somewhat hegemonic society (Schwarz, 2018) where the only perspective is to demonstrate resilience competence in socially acceptable ways, whereas we should on the contrary think about “contextual differences in the definition of what it means to be ‘doing okay’” (Yates et al., 2015, p. 775).

#### Limits Arising From Rutter’s Approach

Additionally, although modern and ecological conceptions of resilience tend to view resilience as a *dynamic* process involving internal and external influences, the significance of such a property is still not quite enough acknowledged. Rutter (2015) demonstrates that too much emphasis is still put on those protective and risk factors. Resilience should rather be “concerned *only* with the differences (albeit large differences) that remain *after* risk and protective factor variations have been taken into account” (Rutter, 2015, p. 342). These factors are neither fixed in their effects nor in what their presence/absence will trigger (Luthar et al., 2015), they are to be considered as nested in and dependent to a context. In the end, the level of stress perceived impacts more the outcome of the resilience process than the “object nature of the [adverse] event itself” (Galatzer-Levy et al., 2018, p. 51).

## Discussion—Towards a Transactional and Constructivist Approach

Having suggested an overall picture of how resilience research evolved towards the current ecological approach, and having attempted to clarify and display concepts, features, and some of the concerns embedded in this field of research, a consensus by the means of a transactional and constructivist approach can now be discussed. The transactional and constructivist approach does not completely replace the previous approaches, but it encompasses them and positions itself as innovative especially because it offers complex conceptual interpretations closely reflecting the human reality.

It should be noted that the authors did not go through the process of coding and reviewing with the idea of transactional and constructivist labeling, these paradigms/terms were noticed in the literature reviewed and were thought to fit well the complex approach next to be described. This section might be considered as a form of conceptual development that stems from the literature reviewed but also goes beyond.

### Core Principles and Theoretical Ground

#### Towards Transactions: Resilience in a Systems-Oriented Perspective

Looking at resilience from a systems perspective could bring understandings further. As research evolves, resilience increasingly draws inspiration from theories of the systems and developmental system theories (Bennett, 2017; Bronfenbrenner & Morris, 2006; Dekker, Bergström, Amer-Wahlin, & Cilliers, 2013; Lerner, 2006). A notable illustration can be found in the transactions some ecosystems engage with their environment in order to face disasters and endure the damaging strokes so that they would adapt and maintain viable functioning (Koffi, 2014; Sivilli & Pace, 2014). In psychology, research is increasingly taking consideration of systems’ understandings to shape the theoretical framework of resilience (Masten, 2007, 2014, 2018; Wright et al., 2013). This shift towards developmental systems theories (DST) in developmental science (Masten & Barnes, 2018) parallels the growing dominance of the trajectory approach in clinical psychology and the positive paradigm shift from deficits-based and individual-level approaches to strength-based and environment-enhancing approaches (Christmas & Khanlou, 2019). All in all these innovations are beneficial to a closer and more nuanced understanding of human reality.

More precisely, Yates et al. (2015) add that the development of an individual is influenced by *transactions* from very different hierarchical levels of the system such as micro, meso, and macro levels. Each level could have stronger or lighter influence, and transactions could even occur between the levels themselves: “Many systems interact or “co-act” to shape the course of development, across levels of function, from the molecular to the macro-levels of physical and sociocultural ecologies” (Masten, 2014, p. 9). Concretely the micro system corresponds to internal characteristics of the individual and peer/family relations, the meso system corresponds to the broader community and institutions such as school, whereas the macro system represents national policies and the cultural context (Bronfenbrenner & Morris, 2006; Thomas, 2005).

Such a system-oriented and transactional perspective could as well evoke the COR theory (Hobfoll, 1989, 2001) advancing that facing adversity triggers transactions of resources, which relates to the individual investing and engaging resources in order to prevent resource loss, to recover from it, or to gain new resources (Hobfoll, 2001).

#### Resilience as a Constructivist and Dynamic Person-Situation-Defined Process

Within the *ecological approach*, competence comes from the individual who *has to* adapt to the environment and to the “*information*” delivered. Precisely in this approach the competence of an individual falls under his or her capacity to adapt to the environment. This aspect is what Hutcheon and colleagues (2013, 2014) were condemning in the above “cripping” critics paragraph. On the contrary, within a *constructivist approach* competence would arise from the actual interaction between the individual and the environment, and would not lie on the individual’s shoulders. Concretely in such an approach the relation between the individual and the environment is perceived in a more complex manner: Competence would rather come from an *equilibrium* between the information released by the environment and the agency of the individual on this environment. Seeing resilience through this lens might then offer opportunities to offset, or at least soften, the current discriminating focus on competence. Currently, several authors such as Ungar and Liebenberg are increasingly turning to constructivist understandings of resilience; they refer to “a person's successful navigation towards the resources necessary to sustain well-being, and the negotiations with those who control resources for these to be provided in ways that are culturally meaningful” (Ungar & Liebenberg, 2013, p. 247). Additionally, although the terminology used mainly remains about “*systems’ thinking*” and “*ecological-transactional*" labels, several other figures of the field also offer complex understandings similar to constructivist reflections (Luthar et al., 2015; Masten, 2015; Panter-Brick, 2015).

Also shared by Pangallo, Zibarras, Lewis, and Flaxman (2015), another crucial matter relates to the person-situation-defined pattern of this conception of resilience. This specificity pertains to the need for resilience to be truly considered as subjective to a situation in which such a process takes place and where both the individual and the context have to be taken into account (Schwarz, 2018). Inter alia, it means that the concept of resilience based on vulnerability, risk, and protective factors could still prevail and function well as long as those notions are renegotiated and rethought depending on the situational context, and on the individual’s profile. According real credit to this specificity could also allow us to keep in mind the subjectivity and contextuality of risk and protective factors’ effects, which would prevent them from being defined simply on the grounds of their nature.

Overall, these dynamic perspectives and situational subjectivity first address that resilience is not “an across-the-board phenomenon” (Luthar et al., 2015, p. 248). Individuals experience resilience in some domains and situations but not in others (Infurna & Luthar, 2018; Luthar et al., 2000, 2015; Rutter, 2013; Wright et al., 2013). Similarly, adversity and protective factors might not affect individuals the same way depending on their subjective sensitivity, or differential susceptibility (Masten, 2014). Two different individuals might not rate risk, adversity, and supportive factors the same way (Bartett, 1994; Luthar et al., 2000).

Second, these perspectives indicate that resilience fluctuates across the lifespan and is highly development-dependent. It might appear to be “ordinary” rather than extraordinary (Masten, 2001)—and *minimal-impact resilience* and *recovery* trajectories may be the most consistently observed (Galatzer-Levy et al., 2018; Infurna & Luthar, 2018)—yet resilience should be considered as never guaranteed (Anaut, 2015). “Although a person responds resiliently to a certain circumstance, new points of vulnerability or strength might appear as a response to change” (Craciun, 2013, p. 4). Hence, time and timing are crucial aspects: 1) *As per* the “temporal issues” deplored by Fisher, Ragsdale, and Fisher (2019) the “time” variable is too often ignored or underestimated even though the *duration, frequency, cycle,* and *rhythm* aspects are crucial elements to consider. 2) Resilience evolves through time points in the lifespan—that is, an individual might experience resilience at some point in life but not at another (Masten, 2014, 2015; Rutter, 2013, 2015; Wright et al., 2013)—and the effects of detrimental experiences or more positive ones on the individual depend on the moment they occur. This is scientifically related to “biological programming effects and the time-varying plasticity of the human brain to lasting effects of both adverse and positive experiences” (Masten, 2015, p. 189)—for example, beneficial effects of early secure mother-infant attachment. All together, the way antecedents occur and express themselves is evolving as the individual develops, so do the mechanisms and consequences.

Third, the acknowledgement of both chronic and acute adversity adds to consideration the possibility for a co-occurrence of either both kinds of adversity or multiple adversities (Hughes et al., 2017). These *piling-up* or *sensitization* effects (Evans, Li, & Whipple, 2013; Fisher et al., 2019; Hobfoll, 2001; Leys et al., 2018; Masten, 2007, 2015)—that is, ongoing or simultaneous occurrence of stressors/adversity—trigger even more diversity in how adversity expresses itself and impacts the individual. Moreover, in regard to the broader context sphere, adversity and factors that foster resilience might have diverse effects depending on *culture* (Masten, 2014; Masten & Barnes, 2018; Noltemeyer & Bush, 2013; Panter-Brick, 2015; Ungar, 2011; Wright et al., 2013). Resilience is not just about doing well in the face of adversity, it is strongly related to cultural norms, moral values, and social aspirations (Noltemeyer & Bush, 2013; Panter-Brick, 2015), so that for example war, violence, enduring, or more general concepts of adversity, resources, positive outcome, good life, aspiration, and relationships, are culturally specific and can therefore bear very different meanings (Liebenberg & Joubert, 2019; Panter-Brick, 2015).

#### A “Measured/Proportional” Understanding of Resilience

Beyond the situational/contextual property mentioned above, resilience could finally be considered in a *measured and balanced* way, it could be “*proportionally*” understood. Adaptative processes occurring in lighter adversity contexts or brief processes of adjustment are sometimes dismissed from the resilience field or poorly considered. Yet those processes in lighter contexts could be proportionally registered under the “resilience” label, because much of the adversity most humans will encounter would more probably constitute lighter disruptions of the every-day life (Davis, Luecken, & Lemery-Chalfant, 2009; Leys et al., 2018). As well Rutter’s “chemistry of the moment” (Rutter, 2015, p. 342) could be debated. He argues that adjustment to lighter or one-time adversity is more about a momentary adaptation rather than resilience. Perhaps this kind of adaptation might seem meaningless when considering resilience under a developmental lens—that is, evolving across the life span, making it a construct the individual builds along his or her development—but according to a balanced and measured design, smaller adjustments to milder stressors could also be held as *constructions* pertaining to some kind of resilience (Liebenberg & Joubert, 2019; Rolin et al., 2018) which might be appraised as part of a greater resilience based on a lifespan perspective.

However, a measured and balanced understanding must not drag resilience towards positivism and simplistic conceptions. The point here is to emphasize the fact that resilience could be suggested regardless of the type, the amount, or the gravity of adversity, as somehow similar patterns of adaptative processes seem to be implied.

### A Definition That Reflects the Complexity of the Construct

Defining resilience is not simply about providing a condensed digest of all the principles discussed previously, it is also about trying to answer the need of consistency (Luthar et al., 2000) and the call for “a generic theory that can be applied across different groups of people and potentially stressful situations” (Fletcher & Sarkar, 2013, p. 17). A complex and nuanced definition could provide researchers with a shared theoretical ground.

From here, within a transactional and constructivist approach, resilience could be referred to as *a person-situation-defined process, referring to the ability of an individual to evidence and draw support from available resources (internal and external) when confronted to adversity. This interaction itself will trigger adaptative mechanisms, therefore enabling the person to face and adjust to that very disturbing or rather challenging adversity. The trajectory of this whole process could either lead back to similar levels of health or drive to improved levels of health. But as we are evolving living creatures, the outcome would anyhow be about a neo-development*.

### Features of the Transactional and Constructivist Approach

The complex approach here at stake does not replace the former features inherent to the traditional and ecological resilience approaches. By looking both subjectively and outwardly (*to an individual and a context*), the transactional and constructivist approach puts the former approaches together to offer a more complex and realistic understanding of the resilience construct. Thereby, traditional and ecological features do not disappear. Yet, in this complex view they cannot be considered as a sum of sporadic and isolated features (Fritz, Fried, Goodyer, Wilkinson, & van Harmelen, 2018). The difference lies—and therefore the progress is held—in the way internal and external antecedents coalesce and interact with each other to influence mechanisms of resilience towards consequences. Disadvantages of both the traditional and the ecological approaches stemmed from the fact they respectively assigned value to either internal psychological characteristics or environmental elements while ignoring to a certain point their complex interactions.

**Figure 4:**
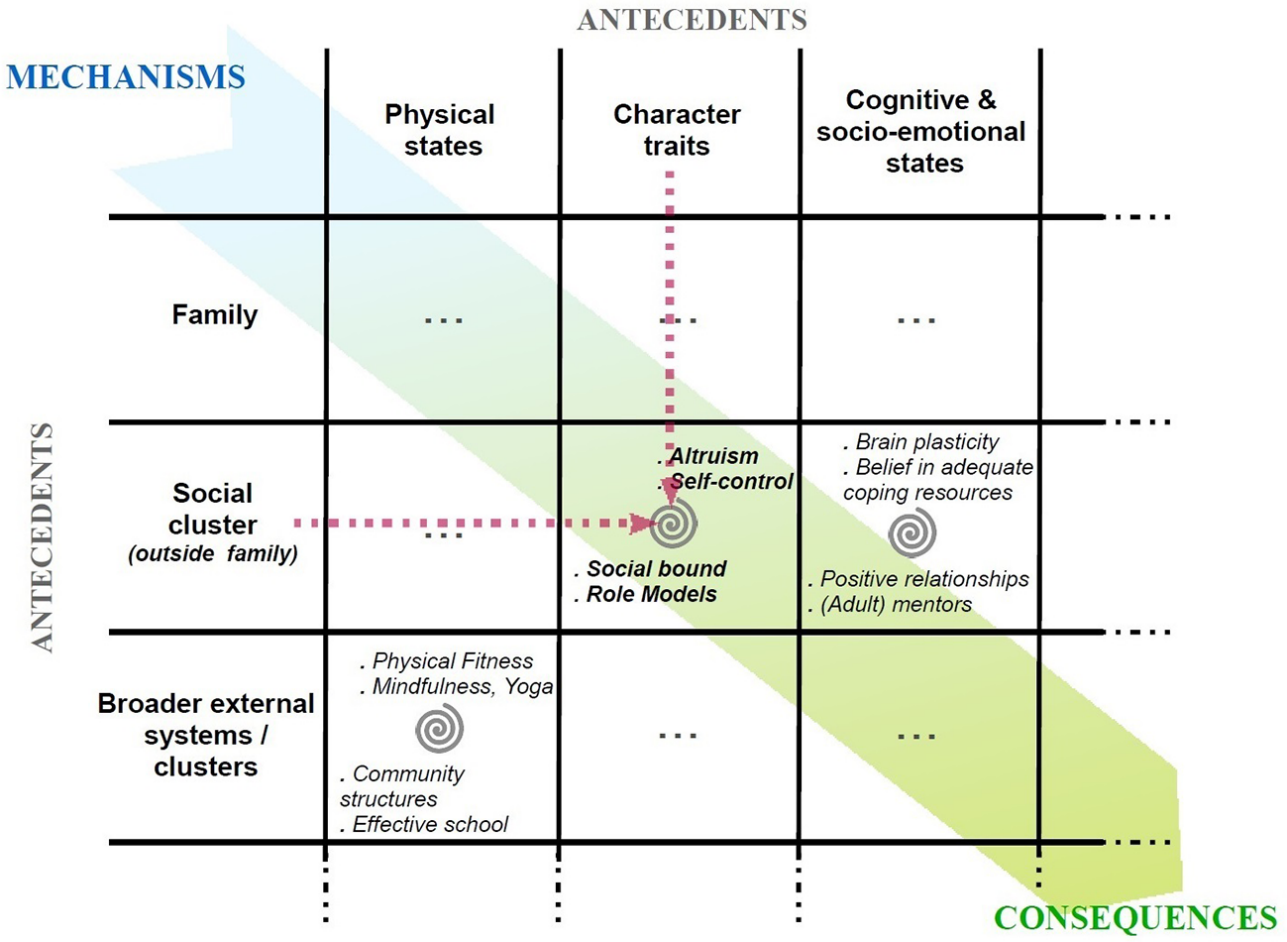
Transactional and Constructivist Approach—Features Agency

Consequently, in the transactional and constructivist approach, features rather operate and behave as dynamic, subjective, and systemic patterns. Clusters might then remain similar (see Tables 2A and 2B in the Supplementary Materials), such as antecedents being divided into “*internal supportive features*” and “*environmental (external) supportive features*,” whereas sub-groups of mechanisms being “*coping*,” “*mentalization/coherence*” processes and “*empowerment*,” to end up with consequences. Yet, Figure 4 below is an illustration of how features of resilience in a transactional and constructivist approach might be processed; they are not listed and clustered separately, they interact and *merge together*. The features agency here is not about a vertical sequence where an antecedent influences another, but on the contrary it is more about a circular alchemy where antecedents and mechanisms modulate one’s perception, sensitivity, and ultimately pervade his or her adaptative functioning.

Where the traditional approach had a very *subjective* eye, and where the ecological approach has a *contextual* eye, the transactional and constructivist approach stands as *integrative* and has a *situational* eye granting the full consideration of a specific individual in an environment and their agency.

### Conclusion

The aim of this integrative review was to examine, to understand, and to synthesize how human resilience is conceptualized within the recent human science literature. The primary research question of this integrative review was: *What are the key approaches, concepts, and definitions of resilience within the recent literature?* Altogether, 69 papers were included in the review and a reference and citation tracking was performed. Besides data abstraction implying a meticulous reading of every paper, definitions and features (antecedents, mechanisms, consequences) of resilience were extracted from every paper. Yet a limitation might be acknowledged: To ensure the review was of a manageable size, four databases were used and the search ranged from 2013 to 2019 (7 years).

As confirmed by several authors and previous reviews, the lack of consensus regarding resilience, as well as the multiplicity of approaches and conceptions present within the recent literature, conceals all the findings relevant to human mental health and therefore calls for clarification. To this end the present paper has focused on the different conceptions, definitions and key approaches to resilience, and has proposed a consensus through the means of a novel and nuanced complex approach. In the first sections of our discourse we have concisely looked at the history of resilience research and at the different waves of its evolution. Going through three authentic controversies, it was then pointed that the ecological approach is a first reaction to the limits of the traditional approach as it illustrates a move from the perspective of an inborn gift or *trait* and an individual subjectivity, to perspectives of a resilience subject to external demands and seen as a process and as a *state*—therefore more dynamic and more educable.

The results section saw the display of all the features involved in the resilience process. These were listed in a quite extensive table and clustered as antecedents, divided into “*internal supportive features*” and “*environmental (external) supportive features*,” whereas mechanisms were divided into “*coping*,” “*mentalization/coherence*,” “*empowerment*,” leading then to consequences. Additionally, the results have highlighted five types of resilience definitions which were then refitted into two broader categories, reflecting whether resilience is about *adapting and bouncing back to previous levels of health and development*, or about *thriving and rising above the adversity towards increased levels of health*.

Yet the ecological broadening of resilience illustrated a switch from the focus on the internal psychological functioning of the individual to a somewhat unequivocal focus on environmental demands. The individual—*no matter the context, the culture, nor his profile*—*has to* adapt to external elements to be considered competent and resilient. Both this overemphasis on competence and this lack of consideration of “*situational*” subjectivity are detrimental issues to the ecological approach as they obscure and devalue natural diversity.

Therefore, in response to the previous approaches, a more complex approach was discussed and proposed as a consensus of all the recent theoretical orientations about resilience. The transactional and constructivist approach brings research forward as it provides a more nuanced and realistic picture of the resilience process which overcomes disadvantages of earlier conceptions. Such an approach may be held as subjective to situations so that it accounts for contextual, inter-individual, and developmental differences. Yet, such as the current and fourth wave of resilience not ignoring previous findings, but rather combining them with technological progress (e.g., neuroimaging techniques), with statistical progress (e.g., growth curve modeling), and with multidisciplinary knowledge, the transactional and constructivist approach, too, tends to appreciate multiple insights and integrates all the diversity embedded in human-environment interactions.

In this complex and nuanced consideration of resilience, competence comes from an equilibrium between the information released by the environment and the agency of the individual on this environment. Here adaptation is not only about the individual’s capacity, it is rather a construction that stems from this complex interaction and from the multiple transactions occurring within and between the levels of a whole system. The *advantage* of this complex approach over the former ones is that where the traditional approach had a very *subjective* eye, and where the ecological approach has a *contextual* eye, the transactional and constructivist approach stands as *integrative* and has a *situational* eye granting the full consideration of a specific individual in an environment and their agency.

Nevertheless, one significant difficulty comes now with field application. As general understandings of resilience become more complex, measures that need to reflect such nuance and complexity shall become as well more complex to implement. In line with this perspective, it could be added that although statistical methodology has evolved, it seems dangerous to make statements about rates of resilience: “It is practically impossible to make definitive ‘*diagnoses of resilience*’ because of the range of plausible adjustment difficulties that must be ruled out” (Infurna & Luthar, 2016, p. 201).

Yet, all in all such a complex approach could be seen as offering an innovative and consistent *pattern matrix* or *framework* for resilience. The idea here would be for researchers and practitioners to rely on a *framework* and an approach that allow complex and deep understanding of the resilience construct, while offering an array of features, agencies, and patterns for any specific field application. Yet, the large scope and complexity of such a framework could allow room for scientists to keep their very specific conceptions and definitions unspoiled. It would enable applications across contexts and situations.

Therefore, in the near future of resilience research, the perspective would be to reflect on field interventions able to focus on supporting one’s internal features and strengths *while supporting at the same time* external features and their beneficial influence. As team resilience and resilience of the team members (see organizational science, workplace) not being the same but still mutually influencing each other (Hartmann et al., 2019), human resilience too is a multidimensional construct where the resilience of individuals, communities, and higher-level institutions may build-up together and influence each other. Interventions must not only target the individual to develop well-being and resilience, but efforts should also encompass the surrounding environments such as resilient families, groups, communities, and institutions via the help of public policies.

## Supplementary Materials

For this article the following Supplementary Materials are available via PsychArchives (for access see Index of Supplementary Materials below).

- Tables 2a and 2b, in which the extracted features of resilience are listed and clustered. These tables represent a more detailed version of Figure 3.
- An Appendix including Tables A1 – A3, which display the literature inclusion and exclusion criteria, the search words, and the list of the papers included in the review.
